# Increased expression of protein kinase CK2α correlates with poor patient prognosis in epithelial ovarian cancer

**DOI:** 10.1371/journal.pone.0174037

**Published:** 2017-03-29

**Authors:** Zebiao Ma, Xiaojing Wang, Jiehua He, Jianchuan Xia, Yanfang Li

**Affiliations:** 1 Sun Yat-sen University Cancer Center; State Key Laboratory of Oncology in South China; Collaborative Innovation Center of Cancer Medicine, Guangzhou, Guangdong, P. R. China; 2 Department of Gynecologic Oncology, Sun Yat-sen University Cancer Center, Guangzhou, Guangdong, P. R. China; 3 Department of Gynecologic Oncology, Cancer Hospital of Shantou University Medical College, Shantou, Guangdong, P. R. China; 4 Department of Gynecologic Oncology, The Affiliated Cancer Hospital of Zhengzhou University; Henan Province Cancer Hospital, Zhengzhou, Henan, P.R. China; 5 Department of Pathology, Sun Yat-sen University Cancer Center, Guangzhou, Guangdong, P. R. China; 6 Department of Biotherapy, Sun Yat-sen University Cancer Center, Guangzhou, Guangdong, P. R. China; University of South Alabama Mitchell Cancer Institute, UNITED STATES

## Abstract

Epithelial ovarian cancer (EOC) is one of the deadly gynecological malignancies. The function of protein kinase CK2α (CK2α) in EOC is still unknown. Our study aimed to investigate the relationship between the protein expression of CK2α and the tumor progression, the prognosis of human EOC. In this study, we analyzed the expression levels of CK2α through Western blot, using EOC cell lines like A2780, HO8910, COV644, OVCAR3, SKOV3, and the primary normal ovarian surface epithelial (NOSE) cells. Furthermore, OVCAR3 and SKOV3 EOC cells were employed as a cellular model to study the role of CK2α on cell growth, migration, invasion, apoptosis, and cell cycle distribution. In addition, we investigated CK2α protein expression in tumor tissues from patients with EOC by immunohistochemistry and analyzed the association between CK2α expression and clinicopathologic parameters and prognosis of EOC patients. And we found that compared with NOSE cells, CK2α protein expression was increased in A2780, HO8910, OVCAR3, and SKOV3 ovarian cancer cell lines. Decreased CK2α expression suppressed OVCAR3 and SKOV3 cell growth and induced more apoptosis. CK2α knockdown using specific siRNAs inhibited migration and invasion ability of OVCAR3 and SKOV3 cells. In addition, high CK2α protein expression was found in 68.4% (80/117) of EOC patients. Increased CK2α expression of was significantly correlated with FIGO staging and peritoneal cytology. Patients with higher CK2α expression had a significantly poorer overall survival compared with those with lower CK2α expression. Multi-variate Cox regression analysis proved that increased CK2α expression was an independent prognostic marker for EOC. Taken together, our data displayed that CK2α may play a role in tumor aggressive behavior of EOC and could be used as a marker for predicting prognosis of EOC patient. High CK2α expression might predict poor patient survival.

## Introduction

Epithelial ovarian cancer (EOC) accounts for approximately 90% of ovarian malignancies [1–3] and is the leading cause of deaths caused by gynecologic cancers. About 70% of patients with ovarian cancer present at an advanced stage [4]. Currently, optimal surgery followed by platinum-based systemic chemotherapy is the standard treatment of ovarian cancer [5–9]. Although the treatment strategies against EOC have been improved over the past three decades [10], the five-year relative survival rate of all stages remains at 45% [11]. Consequently, it is important to explore the biology of EOC and identify new anti-cancer agents.

Protein kinase CK2 is a protein kinase with more than 300 substrates and with multifunction. It consists of two catalytic subunits (CK2a or CK2a’) and two regulatory β subunits [12–15]. CK2 is involved in the processes of cell growth, proliferation and differentiation in normal cells [16]. Studies suggested that CK2α may play an oncogenic role in the development and progression of cancers. In vitro research showed that the knockdown of CK2α resulted in obvious effects on cell proliferation, apoptosis, migration, and the cell cycle [17, 18]. Dysregulations of CK2α have been reported in several solid cancers, including lung [19], breast [20], gastric [21, 22], prostate [23], and bladder cancers [24]. CK2α overexpression has been shown to be a risk factor of poor patient prognosis for several cancers and a potential novel cancer therapeutic target [25, 26].

In ovarian cancer, CK2 was shown to be overexpressed in neoplastic ovarian surface epithelium as compared with normal ovarian surface epithelium [27] and play a role in tumor cell proliferation [28] and apoptosis [29]. It is also overexpressed in ovarian cancer tissues and higher level of CK2α mRNA expression is associated with lower patient survival rate [30]. Although CK2α has been investigated in ovarian cancer cells and in tumor tissues of patient with ovarian cancer, the detailed functional role of CK2α especially in cell invasion and migration in EOC has not been well understood. To better know the role of CK2α in cell activity of ovarian cancer and to investigate the expression of CK2α protein in tumor tissues of Chinese patient with ovarian cancer, We evaluated the effects of siRNA-inhibited CK2α expression on the proliferation, colony formation, migration, invasion, cell cycle, and apoptosis of EOC cell lines and investigated the expression level and prognostic significance of CK2α protein in cancer tissues of Chinese patients with EOC.

## Material and methods

### Cell culture

The human EOC cell lines A2780, HO8910, COV644, OVCAR3, and SKOV3 were cultured in 5% CO2 at 37°C in RPMI 1640 supplemented with 10% (v/v) fetal bovine serum (FBS). OVCAR3 cells were purchased from the China Center for Type Culture Collection (CCTCC, Wuhan, China). SKOV3 and HO8910 cells were obtained from the Shanghai Cell Bank of the Chinese Academy of Science (Shanghai, China), while A2780 and COV644 cells came from Nanjing KeyGen Biotech (Nanjing, China). Primary normal ovarian surface epithelial (NOSE) cells were established according to previous reports [31]. The proteins were extracted from all cell lines in the same culture passage (subculture to 3 passages) for CK2α expression analysis.

### Protein extraction and western blotting

Western blotting analysis was performed to detect CK2α protein levels in cells from 5 EOC cell lines (A2780, HO8910, COV644, OVCAR3, and SKOV3) and NOSE cells, which were extracted using RIPA lysis buffer (Beyotime, Shanghai, China) according to the manufacturer’s protocol. The lysates were cleared by centrifugation (12,000 rpm) at 4°C for 30 min, and the protein concentrations were measured using the BCA Protein Assay Kit (Thermo Fisher Scientific, Waltham, MA, USA). Briefly, equal amounts of protein (30 μg per sample) were separated by 12% sodium dodecyl sulfate polyacrylamide gel electrophoresis (SDS-PAGE), electro-transferred onto a polyvinylidene fluoride (PVDF) membrane (Millipore, Billerica, MA, USA) and subsequently blocked with 5% skim milk in TBST for 60 min. The membranes were incubated overnight at 4°C with rabbit polyclonal antibodies against CK2α (Proteintech, Wuhan, China; 1:1000 dilution) or GAPDH (Proteintech; 1:2000 dilution). After three 10-min washes with TBST, the membrane was then incubated with horseradish peroxidase (HRP)-conjugated secondary antibody (Cell Signaling Technologies, Danvers, MA, USA; 1:2000 dilution) for 45 min at room temperature. After washing, peroxidase activity was detected on X-ray films using an enhanced chemiluminescence detection system (ECL, Cell Signaling Technologies). The band intensity was measured by densitometry using Quantity One software (Bio-Rad Laboratories, Hercules, CA, USA). The target protein levels were normalized with respect to the GAPDH protein levels.

### RNA oligonucleotides and cell transfections

All small interfering RNAs (siRNAs) for the knockout of CK2α were synthesized by GenePharma (Shanghai, China). For our transfection analyses, 2 × 10^5^ cells were seeded in 6-well plates and transfected with siRNA. The four siRNA sequences were as follows: siCK2α#1, sense, 5′-GUGGAUUUAUAGUAGUUCATT-3′ and antisense 5′-UGAACUACUAUAAAUCCACTT -3′; siCK2α#2, sense, 5′-CCUCCCAAAUU- UAGUUCCUTT-3′ and antisense 5′-AGGAACUAAAUUUGGGAGGTT-3′; siCK2α#3, sense, 5′-CCUAAAUCCAACUCAUUUATT-3′ and antisense 5′-UAA- AUGAGUUGGAUUUAGGTT-3′; siCK2α#4, sense, 5′-CCCUUGCUGUGUGUAU- AUATT-3′ and antisense 5′-UAUAUACACACAGCAAGGGTT-3′. For the negative control (siNC): siNC, sense, 5′-UUCUCCGAACGUGUCACGUTT-3′ and antisense, 5′-ACGUGACACGUUCGGAGAATT-3′. Two different siRNAs, siCK2α#1 and siCK2α#3, effectively knocked down the amount of CK2α in the transfected cells. The OVCAR3 and SKOV3 cells were transfected with the indicated siRNAs using the Lipofectamine RNAiMax reagent (Invitrogen) according to the manufacturer’s instructions. The knockdown efficiency was evaluated by western blotting.

### Proliferation assay

The *(3-(4*, *5-dimethylthiazol-2-yl)-5-(3-carboxymethoxyphenyl)-2-(4-sulfophenyl)- 2H-tetrazolium)* (MTS) assay (Sigma-Aldrich, St Louis, MO, USA) was used to measure the growth rates of cells. The cells that were collected after transfection with the indicated siRNAs were plated onto 96-well plates in triplicate at 1 × 10^3^ cells per well. After 24 h, 20 μL of MTS (5 mg/ml) was added to the cells to quantify cell proliferation from 1 to 6 days. The cells were incubated with MTS for 3 h in 5% CO_2_ at 37°C. Finally, the optical absorbance of each well was measured at 490 nm using a microplate reader. Cell growth curves were made by plotting the absorbance (ordinate) against time (abscissa). One independent experiment in triplicate was performed to analyze cell growth.

### Colony formation assay

For the analysis of cell colony formation, transfected cells were routinely harvested, resuspended in complete medium and then placed in 6-well plates (1,000 cells per well). Three control wells were seeded with the same number of cells as the experimental wells. After 10 days of conventional incubation, the surviving colonies were fixed and stained with crystal violet. Colonies that contained 50 or more cells were counted. The colony-forming efficiency (CFE, %) was calculated using the formula: CFE = (colony number/plated cell number) × 100. The experiments were carried out three times independently.

### Cell migration assay

Cell migration assays were carried out using a chamber system consisting of polycarbonate membrane inserts with an 8-μm pore size (Corning, Corning, NY, USA) placed in 24-well cell culture insert companion plates. Cells (5 × 10^4^) in 200 μL of RPMI 1640 containing 5% fetal bovine serum (FBS) were seeded in the upper chamber, and 600 μL of RPMI 1640 containing 15% FBS was placed in the lower chamber at 48 h after the cells were transfected with siRNA. After incubation at 37°C for 24 h, the cells remaining in the upper chamber were removed with cotton swabs. The insert membranes were then fixed with 75% methanol for 30 min, stained with 0.5% crystal violet for 60 min and counted. The stained cells in 10 random microscopic fields per membrane were counted. Each experiment was performed in triplicate.

### Matrigel invasion assay

Matrigel invasion assays were carried out using a chamber system consisting of polycarbonate membrane inserts with an 8-μm pore size (Corning) placed in 24-well cell culture insert companion plates. The inserts were coated with a thin layer of 0.5 mg/ml Matrigel Basement Membrane Matrix (BD Biosciences, Bedford, MA, USA). Briefly, transfected cells were harvested at 48 h and resuspended in RPMI 1640 containing 5% FBS. Cells (4 × 10^5^) in 200 μL of growth medium were added to the upper chamber, and the lower chamber was filled with 600 μL of growth medium containing 15% FBS. After incubation at 37°C for 48 h, non-migrating cells were removed from the upper chamber with a cotton swab. Invading cells on the bottom of the filter were fixed with 75% methanol for 30 min, stained with 0.5% crystal violet for 60 min and counted. The stained cells in 10 random microscopic fields per membrane were counted. Each experiment was conducted in triplicate.

### Apoptosis assay

For the apoptosis assay, cells were routinely collected and centrifuged 72 h after transfection. After washing with cold PBS twice, cells were resuspended in 400 μL of 1× binding buffer and then incubated with 5 μL of Annexin V-FITC (Bestbio) and 10 μL of PI for 15 min in the dark at 4°C. Stained cell numbers were analyzed by flow cytometry (Beckman Coulter). All experiments were performed three times.

### Cell cycle assay

For the cell cycle assay, transfected cells were routinely collected and centrifuged after 48 h. Total cells were washed twice with PBS and fixed with 75% ethanol at -20°C overnight. The cells were then washed in cold PBS, resuspended in 400 μL of PBS containing 20 μL of RNase A and incubated in 37°C for 30 min. Propidium iodide (PI; Bestbio, Shanghai, China) was used to stain cells at 4°C in the dark for 45 min. The cellular DNA content was quantified using a flow cytometer (Beckman Coulter, Brea, CA, USA). All of the experiments were performed three times.

### IHC and semi-quantitative analysis

Paraffin sections were deparaffinized with dimethylbenzene and rehydrated through 100%, 95%, 90%, 80% and 70% ethanol solutions, followed by three phosphate-buffered saline (PBS) washes. For antigen retrieval, the slides were boiled in citrate-hydrochloric acid (pH = 6.0) for 15 min in a microwave oven. Endogenous peroxidase activity was blocked in 0.3% hydrogen peroxide at room temperature for 15 min. After rinsing with PBS, non-specific binding was prevented by 5% sheep serum albumin for 30 min. The tissue sections were then incubated with a rabbit polyclonal antibody against CK2α (Millipore; 1:400 dilution) at 4°C overnight. The tissue section without adding the rabbit polyclonal antibody against CK2α was used as the negative control. After washing, the sections were incubated for 30 min with an HRP-conjugated secondary antibody (Cell Signaling Technology; 1:2000 dilution) at room temperature. Following this incubation, the sections were washed three times in PBS, and the visualization signal was developed with 3, 3′-diaminobenzidine tetrahydrochloride (DAB). All of the sections were then counterstained with hematoxylin. The total CK2α immunostaining score depends on the proportion of positively stained tumor cells and staining intensity, which was determined by two pathologists blinded to the clinical parameters. The proportion of positively stained tumor cells was scored as follows: “0” (<5%, negative), “1” (5%~25%, sporadic), “2” (25%–50%, focal) and “3” (>50%, diffuse). The intensity of staining was graded according to the following criteria: “0” (no staining); “1” (weak staining = light yellow), “2” (moderate staining = yellow brown) and “3” (strong staining = brown). Under an microscope, we selected 10 fields at high power (Magnification: 200×) randomly in each section to score. The total CK2α immunostaining score (ranged from 0 to 9) was calculated by multiplying the two scores of the proportion of positive cells and the intensity of staining. The expression level of CK2α was defined as follows: ‘‘-” (negative, score 0), ‘‘+” (weakly positive, score 1–3), ‘‘++” (positive, score 4–6) or ‘‘+++” (strong positive, score 7–9). CK2α protein expression in EOC tissues was divided into two groups: low CK2α expression group (CK2α‘‘-”or CK2α‘‘+”) and high CK2α expression group (CK2α‘‘++” or CK2α‘‘+++”) [32, 33].

### Tissue sample and patient information

The study was approved by the Ethics Committee of the Sun Yat-sen University Cancer Center. We obtained 117 paraffin-embedded primary EOC samples, which were collected between January 1, 2003 and December 31, 2008, from patients who undergone initial treatment at the Sun Yat-sen University Cancer Center. Serial 2-μm sections from all samples were obtained and used for IHC staining. The histological cell type and stage of tumor tissues were assigned according to the criteria of the World Health Organization (WHO) classification and *International Federation of Gynecology and Obstetrics*, *2009 (FIGO stage*, *2009)*, respectively. Patients’ follow- up consists of outpatient visits and telephone survey. The follow-up included clinical symptoms and laboratory examinations (such as the serum level of CA125 and computed tomography). The overall survival time was measured from initial surgery to the time of patient death or the last follow-up. Patient hospital records were reviewed to obtain demographic data, including the age, serum level of CA125, volume of ascites, tumor size, surgical procedures, FIGO stage, pathological reports, adjuvant chemotherapy, and results of follow-up. The last clinical follow-up data of all patients were May 31, 2014, and the clinicopathological information is summarized in Table 1.

**Table 1:** Relationship between CK2α expression and clinicopathological features of EOC patients.

Characteristics		Number of cases (%)	CK2α expression (%)		*x*^*2*^ value	*P* value
			Low or no expression	High expression		
Age					0.022	0.883
	<50 (years)	40(34.2%)	13(32.5%)	27(67.5%)		
	≥50 (years)	77(65.8%)	24(31.2%)	53(68.8%)		
CA125 (before surgery)					0.18	0.671
	<1000U/ml	70(59.8%)	23(32.9%)	47(67.1%)		
	≥1000U/ml	47(40.2%)	14(29.8%)	33(70.2%)		
Tumour size					0.541	0.462
	<10cm	48(41.0%)	17(35.4%)	31(64.6%)		
	≥10cm	69(59.0%)	20(29.0%)	49(71.0%)		
The volume of ascites					0.285	0.594
	<100ml	28(23.9%)	10(35.7%)	18(64.3%)		
	≥100ml	89(76.1%)	27(30.3%)	62(69.7%)		
Peritoneal cytology ^a^					12.054	0.001*
	Negative	44(45.4%)	22(50.0%)	22(50.0%)		
	Positive	53(54.6%)	9(17.0%)	44(83.0%)		
Pathological type					2.644	0.104
	Serous	63(53.8%)	24(38.1%)	39(61.9%)		
	Others ^b^	54(46.2%)	13(24.1%)	41(75.9%)		
Grade of differentiation ^c^					3.959	0.138
	G1	7(6.9%)	2(28.6%)	5(71.4%)		
	G2	23(22.5%)	10(43.5%)	13(56.5%)		
	G3	72(70.6%)	16(22.2%)	56(77.8%)		
FIGO stage (2009)					11.7	0.008*
	I	13(11.1%)	9(69.2%)	4(30.8%)		
	II	20(17.1%)	5(25.0%)	15(75.0%)		
	III	76(65.0%)	19(25.0%)	57(75.0%)		
	IV	8(6.8%)	4(50.0%)	4(50.0%)		
Lymph node metastasis ^d^					0.574	0.449
	Positive	19(63.3%)	3(15.8%)	16(84.2%)		
	Negative	11(36.7%)	3(27.3%)	8(72.7%)		
Recurrence					2.374	0.123
	Positive	75(64.1%)	20(26.7%)	55(73.3%)		
	Negative	42(35.9%)	17(40.5%)	25(59.5%)		

^a^ In 117 EOC cases, there were 97 records about peritoneal cytology.

^b^ Other pathological types included mucinous adenocarcinoma, endometrioid adenocarcinoma and mixed types.

^c^ Additional five cases were focal cancerous, which had no records about grade of differentiation.

^d^ Only 30 cases underwent retroperitoneal lymphadenectomy.

*P<0.05.

### Statistical analysis

All statistical analyses were carried out using the SPSS statistical software package (version 16.0; SPSS, Inc., Chicago, IL, USA). Survival curves were calculated by Kaplan-Meier analysis and compared using the log-rank test. Correlations between CK2α expression level and the clinicopathological variables were analyzed using the Pearson χ^2^ test. Comparisons between groups were analyzed using Student’s t-test. In addition, univariate and multivariate Cox proportional hazards regression model was used to identify the prognostic effects of clinicopathological factors. All of the tests were two-sided, and *P* < 0.05 was considered to indicate statistical significance.

## Results

### Expression of CK2α protein in EOC cell lines

**Fig 1:**
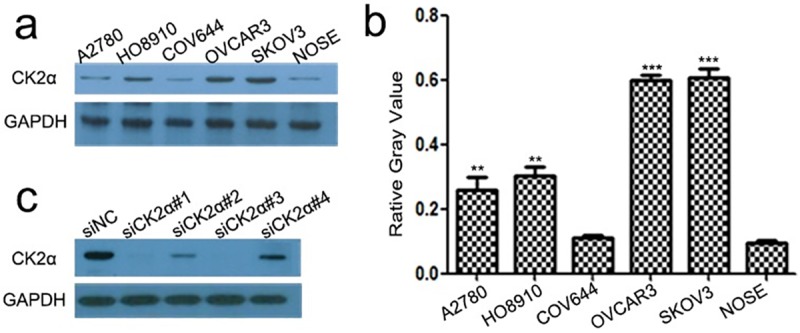
The expression level of CK2α protein in human EOC cell lines as evaluated by western blotting and the knockdown effect by siRNAs against CK2α. (a) CK2α protein was up-regulated in A2780, HO8910, OVCAR3, and SKOV3 cells (particularly in OVCAR3 and SKOV3 cells), but not in COV644 cells, compared with primary NOSE cells. (b) Histogram plotted with six relative gray values from Fig 1A. Each cell line was conducted in triplicate. **P<0.01 *vs*. NOSE; ***P<0.001 *vs*. NOSE. (c) Among the four tested siRNAs against CK2α, siCK2α#1 and siCK2α#3 showed higher knockdown efficiencies in OVCAR3 cells, compared with siNC (NC: negative control).

Five EOC cell lines (A2780, HO8910, COV644, OVCAR3, and SKOV3) were used to examine CK2α expression by western blot analyses. Compared with primary NOSE cells, CK2α protein expression was found to be increased in A2780, HO8910, OVCAR3, and SKOV3 cells (particularly in OVCAR3 and SKOV3 cells, *P*<0.01) but not in COV644 cells (Fig 1A and 1B).

### Inhibition of CK2α expression in EOC cell lines

Western blotting analysis showed a relatively higher expression of CK2α in OVCAR3 and SKOV3 cells than that in other cell lines tested (Fig 1A and 1B). Accordingly, we selected OVCAR3 and SKOV3 cells as the optimal cells to transfect with four CK2α-targeting siRNAs (siCK2α#1, siCK2α#2, siCK2α#3, siCK2α#4) in order to explore the biological function of CK2α in EOC cell lines. Through western blotting analysis, we noted that the CK2α expression levels were markedly decreased in cells transfected with siCK2α#1 and siCK2α#3 compared with those treated with siCK2α#2 and siCK2α#4 (Fig 1C).

### Knockdown of CK2α inhibited EOC cell proliferation in vitro

**Fig 2:**
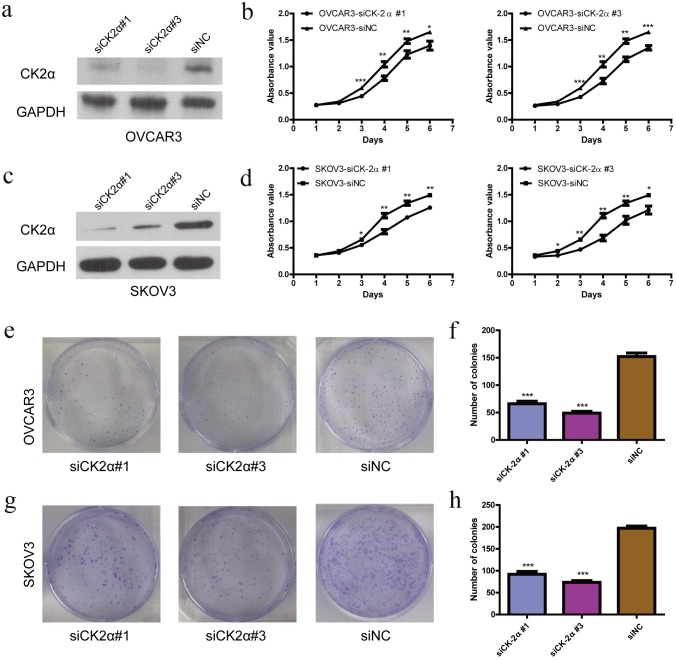
Growth-inhibiting role of knockdown CK2α in OVCAR3 and SKOV3 cell lines. (a, c) The knockdown efficiency of selected CK2α-targeting siRNAs in transfected cells was evaluated by western blotting, and the MTS assay showed that the silencing of CK2α suppressed the proliferation of OVCAR3 (b) and SKOV3 (d) cell lines. (b, d) The difference arose from Day 2 or Day 3 and persisted until Day 6. (e-h) Colony-formation assays indicated decreased growth rates in CK2α-silenced OVCAR3 (e and f) and SKOV3 (g and h) cell lines. One independent experiment was carried out in triplicate. Values are shown as the mean ± standard deviation (SD). Use independent Student's t-test to calculate *P*-values. *P < 0.05 *vs*. the control; **P < 0.01 *vs*. the control; ***P < 0.001 *vs*. the control.

We carried out the cell proliferation and colony formation assay to explore the role of CK2α in the growth of EOC cells. After OVCAR3 and SKOV3 cells were transiently transfected with CK2α-specific siRNAs and siNC RNA for 48 h, they were evaluated in the cell proliferation assays and colony formation assays. The cell proliferation rate in OVCAR3 cells, which were transiently infected with siCK2α #1 and #3, respectively, was notably inhibited compared with that in cells infected with siNC (*P* < 0.05, Fig 2A and 2B), as well as colony-formation abilities (*P* < 0.01, Fig 2E and 2F). Data showed a similar result in SKOV3 cells for both cell proliferation (*P* < 0.05, Fig 2C and 2D) and colony formation (*P* < 0.01, Fig 2G and 2H). These results further supported that CK2α is necessary for the development of ovarian cancer.

### Knockdown CK2α inhibits the migration and invasion of EOC cells in vitro

**Fig 3:**
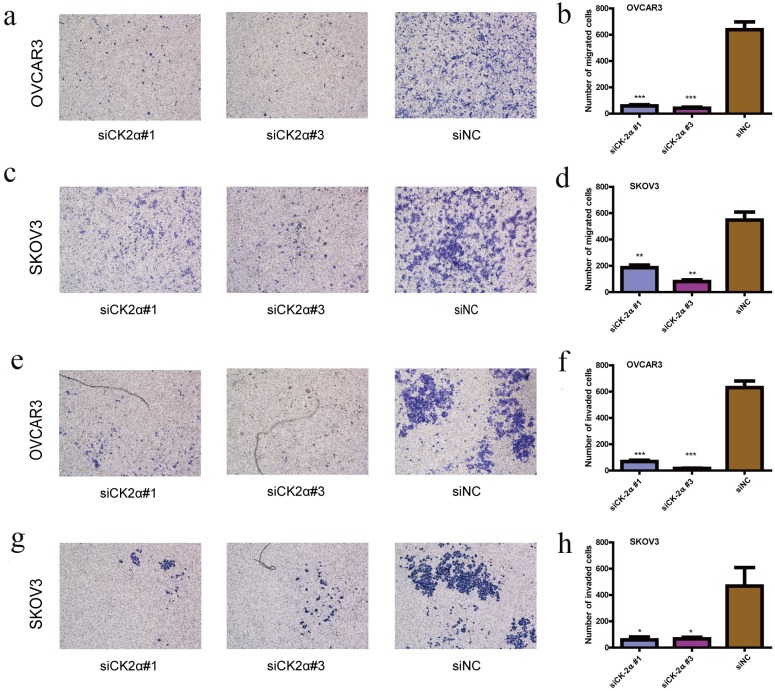
Suppression of EOC cell migration and invasion ability by CK2α silencing. (a-d) CK2α silencing by specific siRNAs inhibited the migration ability of OVCAR3 (a and b) and SKOV3 (c and d) cells in the Transwell migration assay. (e-h) CK2α silencing using specific siRNAs remarkably attenuated the invasion ability of OVCAR3 (e and f) and SKOV3 (g and h) cells in the Matrigel invasion assay. Data are presented as the mean ± SD of three independent experiments. *P*-values were obtained with the independent Student's t-test. *P < 0.05 *vs*. control; **P < 0.01 *vs*. the control; ***P < 0.001 *vs*. the control. Magnification: 200×.

We performed further *in vitro* studies using the transwell migration assay to examine the effect of CK2α on EOC cell motility. Transient transfection of OVCAR3 and SKOV3 cells with siCK2α led to significantly suppressed cell migration and invasion through the membrane in the chamber compared with control cells (Fig 3). Together, these results suggested that up-regulated CK2α expression levels may play an important role for the aggressive characteristics of EOC cells.

### CK2α silencing induced apoptosis, but did not change cell cycle distribution, in EOC cell lines

**Fig 4:**
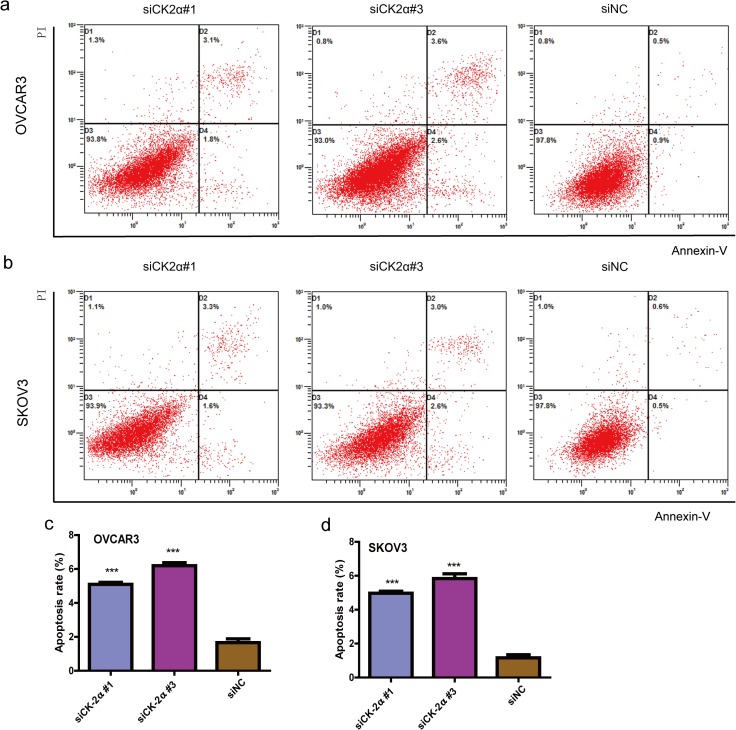
Effect of CK2α silencing on the apoptosis in EOC. Effect of CK2α silencing by siRNAs on the apoptosis of OVCAR3 (a and c) and SKOV3 (b and d) cells. Knockdown of CK2α dramatically induced apoptosis. ***P < 0.001 *vs*. the control.

**Fig 5:**
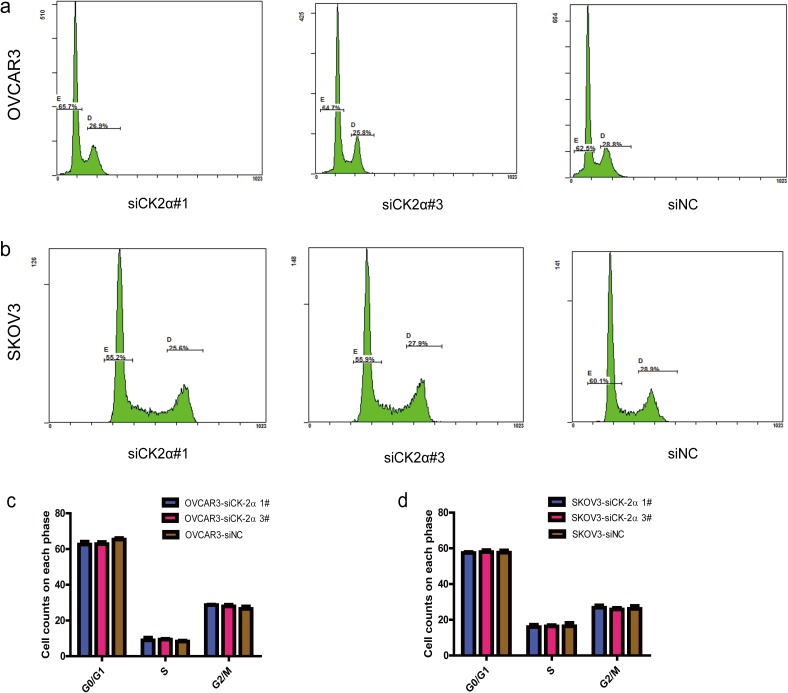
Effect of CK2α silencing on the cell cycle in EOC. There is no significant difference on cell cycle distribution between OVCAR3 (a and c) and SKOV3 (b and d) cells after transfected with CK2α-specific siRNAs and siNC. *P* > 0.5 *vs*. the control.

To investigate whether the CK2α knockdown-mediated suppression of cell growth is associated with cell cycle arrest or the induction of apoptosis, we performed cell cycle and apoptosis analyses by using flow cytometry. Significant differences in Annexin V-positive apoptotic cells based on flow cytometry were observed in the CK2α siRNA-treated cells compared with cells transfected with siNC. Apoptosis was induced in 5.10 ± 0.20% and 6.20 ± 0.30% of the OVCAR3 cells transfected with siCK2α#1 and siCK2α#3, respectively, compared with 1.67 ± 0.38% of those treated with siNC (*P* < 0.001) (Fig 4A and 4C). Similarly, siCK2α#1 and siCK2α#3 induced apoptosis in 4.98 ± 0.21% and 5.83 ± 0.50% of SKOV3 cells, respectively, compared with 1.17 ± 0.31% of those treated with siNC (*P* < 0.001) (Fig 4B and 4D). Cell cycle analysis indicated that the distribution proportions of cells in the G0/G1, S and G2/M phases were not dramatically changed in OVCAR3 (*P* > 0.5 for siCK2α#1 and siCK2α#3) (Fig 5A and 5C) or SKOV3 (*P* > 0.5 for siCK2α#1 and siCK2α#3) (Fig 5B and 5D) cells transfected with CK2α siRNAs compared with those treated with siNC. Our results showed that CK2α may play an important role in EOC development via an anti-apoptotic process.

### Immunohistochemical (IHC) analysis of CK2α protein expression in EOC tissues and its association with patient survival

**Fig 6:**
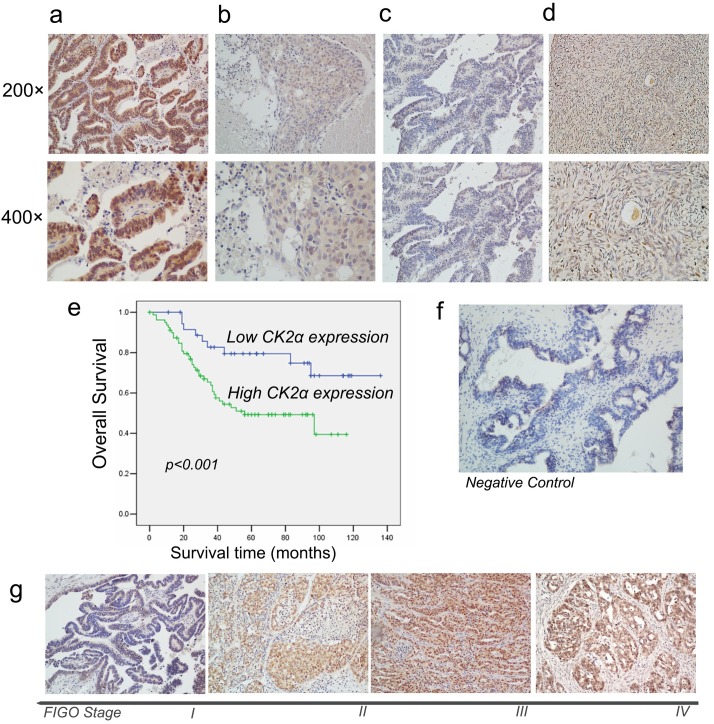
IHC analyses of CK2α protein expression in EOC specimens and Kaplan–Meier survival analyses of the EOC patients (n = 117). (a) Strong CK2α staining in EOC specimens, scored as CK2α (+++). (b) Moderate CK2α staining in EOC specimens, scored as CK2α (++). (c) Weak CK2α staining in EOC specimens, scored as CK2α (+). (d) Low CK2α staining in normal ovarian surface epithelium, scored as CK2α (-). (e) According to CK2α immunostaining score, these 117 EOC patients were divided into two groups, named as low-CK2α expression (n = 37, CK2α- or CK2α+) and high-CK2α expression (n = 80, CK2α++ or CK2α+++) groups. Kaplan-Meier survival curves was plotted to display the survival rate. The five-year survival rate of patients with low-CK2α expression was significantly higher than that of patients with high-CK2α expression (79.5% *vs*. 49.3%, *P* < 0.001, log-rank test). (f) The tissue section without adding the rabbit polyclonal antibody against CK2α was used as the negative control. (g) Samples with a later FIGO stage expressed more CK2α.

To further explore the expression level and prognostic value of CK2α protein in EOC, 117 paraffin-embedded primary EOC tissues confirmed by histopathology were used to perform IHC. The median age of these patients was 52 years with a range of 19 to 83 years, and the median follow-up time was 44 months (range, 0–136 months). In the CK2α-positive specimens, CK2α was detected in the cytoplasm and cell nucleus (Fig 6A–6C). CK2α expression was low in normal ovarian surface epithelium (Fig 6D). High CK2α expression (++ or +++) was found in 80/117 (68.4%) specimens, and low CK2α expression (- or +) was detected in 37/117 (31.6%) specimens (Table 1). The correlations between the clinicopathologic parameters of EOC and expression of CK2α are summarized in Table 1. Chi-squared analyses indicated that CK2α expression level had significant correlation with peritoneal cytology (*P* = 0.001) and FIGO stage (*P* = 0.008), but not with the age, serum CA125 level, tumor size, volume of ascites, pathological type (serous vs. others), grade of differentiation, lymph node metastasis and recurrence (Table 1). Patients with positive peritoneal cytology had a higher score in their samples, which means an increased CK2α expression. Meanwhile, samples with a later FIGO stage expressed more CK2α (Fig 6G). Kaplan–Meier analyses displayed a significant association between high CK2α expression and poor patient prognosis (*P* < 0.001, Fig 6E). The overall survival was significantly higher in the group with low CK2α expression than that in the group with high CK2α expression. Furthermore, univariate and multivariate analyses were also used to evaluate the prognostic role of CK2α expression and other clinicopathologic parameters. Univariate Cox proportional hazard regression analyses revealed that CK2α expression (*P* = 0.010), age (*P* = 0.010), serum CA125 level (*P* = 0.046), volume of ascites (*P* = 0.008), FIGO stage (*P* < 0.001), lymph node dissection (*P* = 0.036), cytoreductive surgery (*P* < 0.001), and peritoneal cytology (*P* = 0.041) were significant overall survival (OS) related factors (Table 2). Multivariate Cox proportional hazard regression analyses showed that both CK2α expression (*P* = 0.030) and FIGO stage (*P* = 0.014) were independent prognostic factors (Table 2). These results indicated that CK2α protein expression level is associated with patient prognosis, and higher CK2α expression predicts poorer patient survival.

**Table 2:** Univariate and multivariate analysis of OS in EOC.

Variables	Univariate analysis			Multivariate analysis		
	HR	95%CI	*P* value	HR	95%CI	*P* value
Age	2.320	1.149–4.685	0.019*	1.209	0.522–2.800	0.658
(≥50 years vs. <50 years)						
CA125	1.805	1.010–3.225	0.046*	1.179	0.585–2.376	0.646
(≥1000 U/ml vs. <1000 U/ml)						
Ascite	2.622	1.110–6.196	0.028*	2.257	0.656–7.768	0.197
(≥100 ml vs. <100 ml)						
FIGO stage (2009)	3.190	1.872–5.437	0.000*	2.407	1.194–4.851	0.014*
Lymph node dissection	0.421	0.188–0.943	0.036*	0.478	0.172–1.325	0.156
Cytoreductive surgery	0.334	0.187–0.599	0.000*	0.548	0.273–1.103	0.092
(>1 cm vs. ≤1 cm)						
Peritoneal cytology	1.982	1.027–3.823	0.041*	0.815	0.391–1.699	0.586
Size	1.024	0.566–1.852	0.938			
(≥10 cm vs. <10 cm)						
Pathological type	1.480	0.829–2.643	0.185			
(Serous vs. Others)						
Grade of differentiation	0.948	0.582–1.544	0.831			
CK2α	2.647	1.268–5.526	0.010*	2.560	1.098–5.970	0.030*
(Low vs. High)						

OS, overall survival; EOC, epithelial ovarian cancer; HR, hazard ratio; CI, confidence interval

*P <0.05.

## Discussion

In this study, we showed that CK2α protein expression was overexpressed in human EOC cell lines through western blotting. CK2α knockdown by using specific siRNAs inhibited cell proliferation, and induced more cell apoptosis. By immunohistochemical staining, high CK2α protein expression was found in the tumors of EOC patients. Moreover, we found knockdown CK2α inhibited the migration and invasion of EOC cells *in vitro*. To our knowledge, there is few published report evaluating the relationship between cell aggressive ability and CK2α in EOC cells. Our data provide new information in this respect. And we first reported CK2α protein expression and its significance in Chinese patients with EOC.

In our data, the expression level of CK2α varies in different ovarian cancer cells. CK2α was over-expressed in four (A2780, HO8910, OVCAR3, and SKOV3, especially in OVCAR3 and SKOV3 cells) of the five ovarian cell lines, but not in COV644 cells, compared with NOSE. Why these ovarian cancer cells had expressed different level of CK2α? One possible explanation is that, EOC has several pathological subtypes, such as serous, endometriod, clear cell, and so on; although the five ovarian cancer cell lines came from patients with ovarian cancer of adenocarcinoma type, these ovarian cancer cells are heterogeneous, may be from different pathological subtypes, and had different biological phenotype. For example, SKOV3 cells are resistant to some cytotoxic drugs including cis-platinum and to totumor necrosis factor [34]. OVCAR3 cells express androgen receptor, estrogen receptor, and progesterone receptor [35]. The exact mechanism underlying why the cells over-express CK2α need to be further investigated.

Ovarian cancer is easy to spread in the peritoneal cavity, which lead to that most patients are diagnosed at advanced stage. In ovarian cancer, previous studies showed that CK2 has a role in tumor cell proliferation [28] and antiapoptosis [29], but no data is available about the role CK2 plays in cell migration and invasion in this cancer. Our functional studies using specific siRNAs showed that silencing CK2α inhibited EOC cell motility and aggression. The results suggested that CK2a is involved in cell migration and invasion and may play a part in tumor development. The findings were consistent with the results of our clinicopathologic analysis, which showed that CK2α overexpression was significantly associated with positive peritoneal cytology and advanced tumor stage. These data suggested that the high expre ssion of CK2α may promote EOC metastasis. Currently, it is generally known that the epithelial- mesenchymal transition (EMT) plays an important role in cancer metastasis. Zou et al. showed that CK2α modulated the cell invasion ability of colorectal cancer cells via regulating EMT-related genes [36]. Furthermore, Rozanov DV et al [37] confirmed that the expression of CK2α was strongly and universally correlated with that of membrane type-1 matrix metalloproteinase (MT1-MMP), which is associated with cancer cell invasion and metastasis. CK2α is identified as one of the specific downstream genes of MT1-MMP.

Previous studies have demonstrated that CK2α plays a role in the apoptosis of human cancers, including ovarian cancer [29], head and neck squamous cell carcinoma [17], glioblastoma [38], and prostate cancer [39]. In what way is CK2 involved in apoptosis? One study showed that CK2 play a role in modulating NF-κB activation, repression of pro-apoptotic TP53 family transcription factors TP53 and TAp63 in head and neck cancer [40]. We also found that the inhibition of CK2α expression significantly promoted the apoptosis of EOC cell lines. This may partly explain the cell growth inhibition caused by siRNA in OVCAR3 and SKOV3 cell lines (Fig 2). Other mechanism may also exist. In lung cancer cells, Zhang S et al. demonstrated the inhibition of CK2α down-regulated Notch1 signaling, which is usually involved in cancer cell proliferation [41]. In ovarian cancer, Chiaramonte R et al. found that the Notch pathway also can promote cell growth via the CXCR4/SDF1α chemokine system [42].

Whether CK2 is involved in cell cycle regulation remains controversial. In malignant peripheral nerve sheath tumors [43], knockdowning CK2 using CX-4945 induced an increase of cells in the G2/M phase of the cell cycles. However, in primary hepatocellular carcinoma [44], silencing of CK2 using specific siRNAs has no effect on proportion of cells in cell cycle. In our study, silencing CK2 using siRNAs did not affect cell distribution in cell cycle. These data indicated that CK2 has different impact on cell cycles in different cancers.

Data in the literature and from our study suggested that CK2α may be regarded as a potential prognostic factor of ovarian cancer. CK2α mRNA was over-expressed in EOC and higher level CK2 expression predicted lower patient survival rate [30]. Using immunohistochemical staining, we showed that CK2α protein over-expression was significantly correlated with more aggressive tumor behavior in terms of positive peritoneal cytology and advanced tumor stage. As of CK2 expression in tumours of different stage, it is higher in tumors of stage II and III compared with that in stage I (75%, 75% *vs*. 30.8%), but the expression in stage IV is a little lower compared to that in stage III (50% *vs*.75%). This may be due to the small sample (n = 10) in stage IV group. Further analysis showed that EOC patients with higher CK2α expression had significantly shorter OS than those with lower CK2α expression. Multivariate analyses revealed that CK2α expression was an independent prognostic factor for patient survival. These results suggested that CK2α protein over-expression is predictive of poor patient survival.

## Conclusion

In conclusion, CK2α may play a role in the development and progression of EOC and could be used as a tumor marker for the prognosis of EOC patients. High CK2α expression might predict poor patient survival.

## Supporting information

S1 Table Gray value of cells for Fig 1B. (XLS)

S2 Table Data of proliferation assay for Fig 2B and 2D. (XLS)

S3 Table Data of colony formation assay for Fig 2F and 2H. (XLS)

S4 Table Data of cell migration assay and matrigel invasion assay for Fig 3B, 3D, 3F and 3H. (XLS)

S5 Table Data of apoptosis assay for Fig 4C and 4D. (XLS)

S6 Table Data of cell cycle assay for Fig 5C and 5D. (XLS)

S7 Table Clinical data for Tables 1 and 2, Fig 6E. (XLS)
